# Lack of effect of deep brain stimulation on resting-state functional connectivity in essential tremor provides valuable insights

**DOI:** 10.1093/braincomms/fcae060

**Published:** 2024-02-28

**Authors:** A M Madelein van der Stouwe

**Affiliations:** Department of Neurology, University Medical Center Groningen, 9700 RB Groningen, the Netherlands; Expertise Center Movement Disorders Groningen, University Medical Center Groningen, 9700 RB Groningen, the Netherlands

## Abstract

This scientific commentary refers to ‘Deep brain stimulation does not modulate resting-state functional connectivity in essential tremor’, by Awad et al. (https://doi.org/10.1093/braincomms/fcae012).

This scientific commentary refers to ‘Deep brain stimulation does not modulate resting-state functional connectivity in essential tremor’, by Awad et al. (https://doi.org/10.1093/braincomms/fcae012).

A publication bias exists against the publication of negative findings in neuroimaging experiments,^1^ while these studies can be of great value for the scientific advance in a study field. Factors impeding publication can be found on the part of the authors, who may lose interest in their experiment or may feel the results will reflect negatively on their skill; on the part of the reviewers, who may find it easier to find fault with the methodology of papers reporting negative results; and on the part of editors, who may think such papers will get less citations, reflecting negatively on their journal. Luckily, some papers find their way through this potential minefield: a case in point is the paper ‘Deep brain stimulation does not modulate resting-state functional connectivity in essential tremor’, by Awad et al., published recently in *Brain Communications*.^2^ The negative results reported here provide clues for our concept of essential tremor pathophysiology and for the methodology of future tremor studies.

Essential tremor is the most common movement disorder, and while our understanding of this debilitating disorder has increased in the last decades, its exact pathophysiology as well as the mechanism underlying established treatments such as deep brain stimulations (DBS) is not yet understood. Neuroimaging techniques can help to identify the pathophysiological mechanisms causing tremor. Functional neuroimaging, especially functional MRI, has been particularly useful in establishing essential tremor as resulting from pathological oscillations within the cerebello-thalamo-cortical circuit.^3^ Essential tremor may result from increased, pathological cerebellar activity driving the network^4,5^ (cerebellar oscillator hypothesis), or essential tremor may be caused by cerebellar disconnection from the network (cerebellar decoupling hypothesis).^6,7^

Investigating essential tremor patients implanted with DBS allows the investigation of both pathological activity and its response to treatment. Such an DBS-ON versus DBS-OFF paradigm was used by Awad et al. in their article in *Brain Communications*,^2^ while scanning patients at rest. This paper is a worthwhile companion to their previous study,^8^ in which they explored the influence of DBS in the caudal zona incerta on the cerebello-thalamo-cortical circuit during a tremor-inducing postural task. While patients held this posture, switching on DBS was associated with decreased cerebral activity in the primary sensorimotor cortex and cerebellar lobule III. These effects of DBS are located within the sensorimotor cerebellar lobules, in which previous studies have shown increased tremor-related activity,^4^ and can therefore be interpreted as a normalization of cerebellar overactivity through DBS treatment, befitting the cerebellar oscillator hypothesis.

In the current paper,^2^ the authors report on the effect of DBS while patients are at rest rather than during movement, a logical next step. Given the known changes in resting-state brain connectivity in essential tremor, it is an interesting question whether these changes are affected, or indeed reversed or normalized, by DBS. In a previous meta-analysis of resting-state functional connectivity, decreased functional connectivity of cerebellar lobule IV–V was indicated as the hallmark of tremor in comparison to healthy participants.^9^ More specifically, in essential tremor patients, decreased functional connectivity has been reported between the dentate nucleus and cortical, subcortical, and cerebellar areas compared to healthy participants, while these changes correlated with tremor amplitude, severity and disease duration,^6^ fitting with cerebellar decoupling. Given these findings, the authors expected that DBS would modulate the functional connectivity within the cerebello-thalamo-cortical circuit during rest.

However, despite a truly thorough analysis to establish changes in resting-state functional connectivity ON versus OFF DBS, the authors did not find any in their cohort of essential tremor patients. Of course, as is mentioned by the authors, the study’s sample size is relatively small. But given the challenges in including patients with an implanted DBS system into an MRI study, the sample size is reasonable, and based on the distribution of correlations shown, it seems unlikely that including more patients would have led to a different result. As such, this negative result should be valued.

Comparing the clear effect of DBS on activity within the cerebello-thalamo-cortical circuit during movement with the lack of effect of DBS on connectivity during rest, the authors conclude that DBS modulation of the sensorimotor circuit in essential tremor is action-dependent. This indeed seems plausible in a movement disorder that is characterized by action tremor, and in which DBS is an effective symptomatic treatment. Note that only seven of the investigated patients had a mild tremor at rest, while patients with severe resting tremor were not included because of the risk of motion artefacts. Most patients will consequently have shown no active symptoms during the resting-state scan. This makes one wonder whether functional connectivity is in fact altered by DBS in patients with resting tremor, for instance in tremor dominant Parkinson’s disease. This seems to be the case as normalization of functional connectivity by switching on subthalamic DBS has previously been reported in patient with Parkinson’s disease, although ‘excessive tremor at rest that would make scanning impossible’ was an exclusion criterion in this study.^10^ A direct comparison between tremor dominant Parkinson’s disease and essential tremor patients would help substantiate the hypothesis that DBS affects functional connectivity differently in tremor manifesting at rest and during action. Moreover, researchers working on essential tremor may want to be mindful that while functional MRI resting-state has seen substantial growth in previous years, the results reported by Awad et al. reflect that it may not be the most relevant state for investigation in essential tremor.

As such, this new paper with its negative results has provided us with insights into the mechanism behind the symptomatic treatment of essential tremor by means of DBS, and with inspiration for future work.
